# How to Make Medical Device Registry Data Fit for Purpose; A Response to Recent Commentaries

**DOI:** 10.34172/ijhpm.10150

**Published:** 2026-07-22

**Authors:** Lotje A. Hoogervorst, Rob G.H.H. Nelissen, Tom Melvin, Anne Lübbeke, Alan G. Fraser, Stefan James, Perla J. Marang van de Mheen

**Affiliations:** ^1^Department of Orthopaedics, Leiden University Medical Center, Leiden, The Netherlands.; ^2^Trinity College Dublin, Dublin, Ireland.; ^3^Division of Orthopaedic Surgery and Traumatology, Geneva University Hospitals and University of Geneva, Geneva, Switzerland.; ^4^Department of Cardiology, University Hospital of Wales, Cardiff, UK.; ^5^Department of Cardiology, Uppsala University, Uppsala, Sweden.; ^6^Department of Safety & Security Science, Delft University of Technology, Delft, The Netherlands.

 Three commentaries have recently been published in response to our manuscript “Quality and Utility of European Cardiovascular and Orthopaedic Registries for the Regulatory Evaluation of Medical Device Safety and Performance Across the Implant Lifecycle: A Systematic Review.”^1-4^ Our review identified much heterogeneity and limited public transparency regarding the structural and methodological characteristics of the registries that were reviewed, which implies that it is currently challenging to pool data across registries, and to judge the regulatory utility of performance and safety data reported by registries.^1^ The commentaries provided valuable perspectives on how the regulatory utility of registry data can be strengthened.^2-4^

## Data Quality and Limitations of Pooling Data

 The use of registry data for regulatory decision-making is limited by variations in how data are collected, defined, analysed, interpreted, and reported. All commentators acknowledge these goals as important challenges that extend also beyond the European context. They propose several approaches to improve the utility of registry data, including (*i*) coordinated registry networks to enhance harmonisation and comparability of registry data,^2^ (*ii*) linkage with complementary real world data sources such as electronic health records,^4^ and (*iii*) the use of federated analytical approaches and common data models as done in the European Health Data and Evidence Network.^4^ We agree that all these developments are important.

 Even with harmonised or linked datasets and with federated analyses, however, regulators will need to evaluate the quality of the real-world evidence and whether these are “fit for purpose,” meaning validated data on implant performance and safety, including the quality of the underlying (linked) data and what constitutes a good-quality federated analysis. Within the Coordinating Research and Evidence for Medical Devices (CORE-MD) project, therefore, we convened a Delphi panel of 51 participants from a broad range of stakeholders to develop a decision framework for regulators. Twenty-seven items were proposed across four domains: data suitability for regulatory assessment, data governance, data quality, and data analysis.^5^ Additional items could be included specific to linkage or federated analyses. Quality of data (eg, completeness) as well as quality of analysis (eg, well-defined outcome measures) were considered most important. Our framework could now be considered when regulators determine how to evaluate and approve evidence from device registries.

 Coordinated registries with harmonised data fields will indeed facilitate pooling of outcomes across countries, thereby increasing statistical power to detect any safety concerns early, and providing evaluation of implant performance across diverse settings. As exemplars, the International Society of Arthroplasty Registries has developed frameworks for collaborative activities and provides a network for established and developing registries.^6^ Numerous international registry studies using individual or aggregate data have been conducted and published, which was possible because of increasing harmonisation of data fields. Similarly, the European Society of Cardiology has developed a growing European registry programme (EuroHeart) as a collaboration between established and new national registries, covering common cardiac diseases such as coronary syndromes, valvular heart disease and heart failure, as well as procedures such as percutaneous coronary and valve interventions.^7^ Registration in EuroHeart is based on published lists of 70–100 standardised mandatory variables per disease, to harmonise data and outcomes. Having mandatory variables is important as pooling registry data that are incomplete will exacerbate any bias.

 This approach will be valid only for implants that are used across multiple countries. In another study, we found that only 68% of total knee prostheses were reported in at least two registries and in sufficient numbers, meaning that for a substantial subset of implants evidence may be available only from an individual registry.^8^ Furthermore, if variants of a single prosthesis are lumped together within a registry, then unsatisfactory outcomes with one variant may be obscured by satisfactory performance of other variants used in larger numbers. This phenomenon, called camouflage, implies that registry data need to be reported with considerable granularity and interpreted in detail by experts.

 The performance of an implant also depends on surgical factors (eg, experience with that specific implant and its instrumentation, surgical learning curves) and patient characteristics.^9^ We reported previously how registry data from different countries showed different performance for the same orthopaedic implant, likely due to differences in the indication for revision.^10^ Performance in diverse settings may better reflect real-world outcomes, but it can be questioned if data should be combined when there are significant regional variations in surgical practices, such as a systematic lower tendency of surgeons to revise a less optimally performing implant in a country. This would reduce the value of evidence because interpretation of the pooled estimate as an indicator of device performance then becomes complicated.

## Utility and Transparency of Registry Data for Different Stakeholders

 As indicated by Mauch, transparency in reporting is essential for building trust and ensuring accountability; if results are reported in detail, then registry data can provide valuable insights for all stakeholders.^2^ Clinicians, manufacturers, regulators, and patients need different information. Regulators and manufacturers want to be sure if any deviation in performance can be attributed to the implant (eg, related to material or design), rather than to surgical or patient factors, before they perform a corrective action. Pooling data can help in this context to detect such problems as early as possible. Patients on the other hand want to be assured not only that the design and material are safe, but also that the implants they receive are appropriate for their specific condition and safe to be implanted by surgeons. Thus, they will be more interested in data from their hospital within such a registry rather than a pooled estimate. Whether registries should invest in common data models, harmonised definitions, and coordinated governance structures, may thus depend on the primary stakeholders that the registry aims to serve, even though ideally a medical device registry should be designed collaboratively so that it can meet the needs of the different stakeholders.

## The Role of Registries for Pre-market Evaluation and Post-market Surveillance

 As shown by the CORE-MD project and emphasised in the commentary by Holtzman and Redberg,^3^ many new high-risk medical devices enter the market with relatively limited clinical evidence so to detect safety issues we rely on registries and the reporting of adverse events during post-market surveillance.^11^ This is particularly relevant for high-risk medical devices in the European Union (EU), as proposed legislative changes would reduce the clinical evidence required for high-risk devices by promoting the use of data from other “similar” devices (known as “equivalence”).^12^ As a result of this, new high-risk implants may enter the market without any clinical evidence. Rules concerning transparency of clinical evidence (clinical investigation reports, Medical Devices Regulation [MDR] Article 77(7); and summaries of safety and clinical performance, MDR Article 32(2)) have been subject to partial implementation, and as a result, it may not be possible to access the clinical evidence for high-risk implants in practice. Consequently, non-regulatory sources such as registry data or published studies may be the only source of clinical evidence to guide selection of implants.

 Published opinions of the expert panels under the MDR (who review the notified bodies’ assessments of novel and high-risk devices) demonstrate that expert panels disagreed with notified body assessments in the majority of published opinions and criticised the lack of methodological rigor of studies underlying the clinical evidence.^13^ This underscores the importance of scientific clinical evidence, which the legislation is meant to support. Previous CORE-MD recommendations provided guidance on clinical study designs and could be extended by more specific guidance on eg, minimum sample size or linking the design and quality of pre-market studies to post-market requirements to generate sufficient quality evidence.^14^ Where registries are widely recognised as an important data source for post-market surveillance, they could be used more effectively if linked to the pre-market evidence for a given implant. This will be of increasing importance to the EU regulatory framework, as periodic reassessments by notified bodies are proposed to be removed from the MDR in all but exceptional cases.^12^ The EU regulatory framework has not yet developed approaches to balance pre- and post-market evidence requirements.

 Registries could also be used to assess the expected impact of small iterative changes in the design or material of an implant. This would help to address current challenges for manufacturers in determining whether a clinical investigation is needed for changes in design, and for notified bodies in deciding whether or not a modification to a device needs to be supported by new clinical evidence. There are examples of orthopaedic implants which manufacturers have considered to be equivalent, where their new design variants have put patients at risk.^11^ Within the EU and when there is uncertainty, notified bodies have the option of issuing a certificate of conformity (equivalent to CE marking) with the stipulation that more data must be collected in the post-market phase, but that has been rarely applied.^15^ It is similar to conditional approval or coverage with evidence development, as practised in other jurisdictions. Registries could play an important role in this context by estimating the expected performance of a new implant variant based on existing evidence from comparable implants. Importantly, these predictive assessments could quantify the remaining uncertainty in expected performance, thereby informing the design of post-market studies or when we may expect to have sufficient additional evidence from registries to ensure that an implant is safe. A broader question then is how much uncertainty is acceptable, and who should determine this. Medical devices are not held to the medicines standard of “safety and efficacy” and as a result, defining and setting requirements for acceptable levels of uncertainty is inherently complex – uncertainty is not solely a statistical issue. Further complexity is added by the polycentric nature of the regulatory framework for medical devices in the EU, and the lack of a forum for scientific development of regulatory requirements. Different stakeholders may have different risk tolerances—for instance, some patients may accept greater uncertainty to get access to potentially beneficial new implants, whereas regulators and clinicians may require stronger evidence.

## Conclusion

 The value of high-quality registry data is paramount, but our systematic review and the published commentaries demonstrate that their regulatory utility can be improved. For that objective, coordinated registries, data linkage, data pooling, and federated analyses, are all important. We can be optimistic about the continuing development of medical device registries and their integration into regulatory processes, but ultimately their success will depend on whether their data are fit for purpose, endorsing safe implants for patients.

## Acknowledgment

 The CORE-MD project was supported by Coordination and Support (CSA) grant 965246 from the Horizon 2020 programme of the EU.

## Disclosure of artificial intelligence (AI) use

 Not applicable.

## Ethical issues

 Not applicable.

## Conflicts of interest

 Authors declare that they have no conflicts of interest.
